# Dynamic Banking Systemic Risk Accumulation under Multiple-Risk Exposures

**DOI:** 10.3390/e24121848

**Published:** 2022-12-19

**Authors:** Hong Fan, Miao Tang

**Affiliations:** Glorious Sun School of Business and Management, Donghua University, Shanghai 200051, China

**Keywords:** banking systemic risk, interbank lending market risk, entity industry credit risk, market risk, Δ*CoVaR*

## Abstract

Much of the existing research on banking systemic risk focuses on static single-risk exposures, and there is a lack of research on multiple-risk exposures. The reality is that the banking system is facing an increasingly complex environment, and dynamic measures of multiple-risk integration are essential. To reveal the risk accumulation process under the multi-risk exposures of the banking system, this article constructs a dynamic banking system as the research object and combines geometric Brownian motion, the BSM model, and the maximum likelihood estimate method. This article also aims to incorporate three types of exposures (interbank lending market risk exposures, entity industry credit risk exposures, and market risk exposures) within the same framework for the first time and builds a model of the dynamic evolution of banking systemic risk under multiple exposures. This study included the collection of a large amount of real data on banks, entity industries, and market risk factors, and used the ΔCoVaR model to evaluate the systemic risk of the China banking system from the point of view of the accumulation of risk from different exposures, revealing the dynamic process of risk accumulation under the integration of multiple risks within the banking system, as well as the contribution of different exposures to banking systemic risk. The results showed that the banking systemic risk of China first increased and then decreased with time, and the rate of risk accumulation is gradually slowing down. In terms of the impact of different kinds of exposures on system losses, the credit risk exposure of the entity industry had the greatest impact on the banking systemic risk among the three kinds of exposures. In terms of the contribution of the interbank lending market risk to the systemic risk, the Bank of Communications, China Everbright Bank, and Bank of Beijing contributed the most. In terms of the contribution of the bank–entity industry credit risk to the systemic risk, the financial industry, accommodation and catering industry, and manufacturing industry contributed the most. Considering the contribution of market risk to the systemic risk, the Shanghai Composite Index, the Hang Seng Composite Index, and the Dow Jones Index contributed the most. The research in this paper enriches the existing banking systemic risk research perspective and provides a reference for the regulatory decisions of central banks.

## 1. Introduction

In financial systems, more than 80% of financial assets are bank assets, and banks play a decisive role. Therefore, to maintain the stability of financial systems, we need to supervise and focus on the banking sub-systems within them. Generally, the monitoring and supervision of banking systemic risk have been mainly based on the study of a single risk, where each type of risk is unrelated to the other and measured separately [1,2]. However, with in-depth research, it has been found that this is not the case. The complex banking system is exposed to multiple types of risks, such as interbank lending risk [3,4], entity industry credit risk [5,6], market risk [7,8,9], and so on. There is an intricate relationship between various types of risks, which tends to amplify or reduce banking systemic risks, significantly affecting the accuracy of bank risk measurement results [10,11]. Different risk factors impact the banking system, causing a chain reaction in the banking system and thus generating a systemic crisis. Therefore, it is very meaningful to measure the contribution of different risk exposures to the systemic risk of banks. This helps regulators identify important risk exposures and develop reasonable supervisory programs to prevent systemic risk at the source.

The ΔCoVaR approach [12] captures the marginal degree of contribution of different risk factors to the overall systemic risk, and with its advantages of simplicity and logical intuition, it has now been widely used in measuring the contribution of systemic risk. Brunnermeier et al. [13] used the ΔCoVaR approach to quantify the contribution of financial institutions to the overall level of systemic risk and investigated the relationship between bank asset price bubbles and systemic risk. Chen et al. [14] measured the systemic correlation between a single bank and the banking system in China and the systemic correlation between any two banks based on the asymmetric ΔCoVaR approach and found that the two main influencing factors of systemic correlation are the characteristic variables of the banks themselves. Banulescu-Radu et al. [15] extended the ΔCoVaR approach and marginal expected shortfall approach, proposed a systemic risk measure for identifying the financial institutions that contribute the most to the overall risk of the financial system, and evaluated the future inferred from an empirical study of U.S. financial institution indicators of early warning systems for systemic crises. However, previous studies that used the ΔCoVaR approach to quantify the contribution of different exposures to banking systemic risk were not found.

For this reason, the main contributions of this paper are as follows: First, due to the lack of existing research on banking systemic risk with integrated multiple exposures, this paper constructs an integrated framework with interbank lending market exposures, entity industry credit exposures, and market exposures. Based on the geometric Brownian motion, the BSM model, and the maximum likelihood estimate method, we studied the dynamic evolvement laws of banking systemic risk under multiple exposures. Second, the lack of data is also a widespread problem in bank systemic risk research. We collected a large amount of bank, entity industry, and market risk factor data, and we combined the ΔCoVaR approach to discuss the degree of contribution of different exposures to banking systemic risk. Finally, the contributions of specific banks, entity industries, and market risk factors to systemic risk were located in the three exposures to investigate their impact on banking systemic risk.

The remainder of this paper is organized as follows: Section 2 reviews the work related to this study; Section 3 presents a model of the dynamic evolution of banking systemic risk under multiple-risk exposures; Section 4 presents the empirical analysis of the collected data; and Section 5 consists of conclusions and future work.

## 2. Literature Review

Most of the current research on banking systemic risk focuses on single-risk exposure. First, most of the results concern interbank lending market exposures, where interbank lending provides banks with liquidity facilities while also providing them with a channel for risk accumulation [3,4,16,17,18]. Since Allen and Gale [19] used the static network structure of the interbank lending market to study the risk contagion of the banking system, many studies on the exposure of the interbank lending market have been enriched. Glasserman and Young [20] studied the evolutionary mechanism of the impact of the interbank default contagion on the banking network parameters and found that external asset losses directly affect the probability of bank default. Gao and Fan [21] studied the macroeconomic impact on the stability of interbank lending networks. Sun et al. [22] studied the performance of the interbank lending market and housing market conditions during two banking crises and found that interbank lending relationships had a greater impact on systemic risk during banking system crises. Huang et al. [23] compared the differences in the structural characteristics of interbank lending market networks constructed by the maximum entropy and minimum density methods and the risk contagion results under the two networks and found that the bank failure risk contagion of the interbank market network based on the minimum density method was wider and stronger. Mitchener and Richardson [24] found that during the Great Depression, panic motivated banks to pull funds from lending banks, and their mutual lending networks amplified systemic risk and eventually caused credit contraction.

Secondly, some research results concerned the market risk exposure faced by the banks [7,8]. Keppo et al. [25] analyzed a bank operating under Basel credit and studied the market risk requirements to maximize its value through recapitalization, dividends, and investment in liquid assets. Diebold and Yilmaz [26] provided a method for calculating volatility spillover based on the VaR model, and further calculated the spillover and spillover effects of four market risk factors, namely foreign exchange, stocks, bonds, and bulk commodities, in the U.S. market. Wu et al. [27] found that the leverage effect of Chinese stock market yields is asymmetric and varies in terms of time. Wang et al. [28] constructed a heterogeneous volatility spillover–generalized autoregressive conditional heteroskedasticity model to study the volatility spillover in the U.S. stock market and found that there was a significant risk spillover from the U.S. stock market to the five stock markets of Japan, France, Canada, the U.K., and Germany; this risk spillover would be more significant during a recession. Martins et al. [29] showed that there is a positive relationship between bank stock returns and entity estate returns after controlling for general market conditions and interest rate changes. El-Massah et al. [30] studied the exchange rate risk of the banking industry in central and northeast Africa, and their findings indicate that the magnitude of the impact of exchange rate risk on banks is significantly associated with the type of bank. He et al. [31] used regressions and systemic risk indices to study risk propagation among Chinese financial markets and concluded that the focus of the prevention and control of Chinese financial systemic risk should be on the stock and fund markets. Chen et al. [9] studied the risk contribution of oil and the USD–RMB exchange rate to the Chinese stock market based on the ΔCoVaR approach and found that oil has a greater impact on the Chinese stock market; however, the USD–RMB exchange rate has a higher contribution during the period of China’s exchange rate system reform.

Finally, in the study of credit risk exposure between banks and entity industries, Elsinger et al. [32] studied the impact of interbank lending market risk and common exposures faced by banks on banking systemic risk separately and found that common exposures faced by banks are more influential on banking systemic risk than interbank lending market exposure. Sun et al. [33] studied the association between interbank contagion risk and entity estate loan losses, and when there is a link between entity estate loans and banks, losses can trigger interbank contagion. Li et al. [34] constructed a bank and firm systemic risk model based on the debt hierarchy approach, studied bank and firm systemic risk and contagion effects in China in 2018, and explored the impact of credit policy easing on maintaining the stability of the banking and corporate credit system. Silva et al. [35] assessed systemic risk and found that feedback from the entity and financial sectors is important and that most models that do not take these factors into account, which can seriously underestimate systemic risk. Degryse et al. [36] proposed a demand control model and studied the risk-taking of banks and firms in credit shocks using data from Belgian banks for the period 2002–2012; they found that when banks are subjected to a large credit shock and have a lending relationship with firms at the same time, then firms are severely negatively affected, which affects their profitability. Li et al. [5] studied the two-tier credit network of bank firms and the bank firm co-financing relationship based on the DebtRank approach and found that assets were positively correlated with DebtRank values and that firms had a greater impact on the banking systemic risk. Wang et al. [6] analyzed the impact of the nonfinancial industry on the financial system based on default clustering and the Δ*CoVaR* approach and found that the manufacturing, wholesale and retail, and real estate industries were highly correlated with systemic risk.

In summary, with the continuous in-depth research on bank systemic risk, there is a large amount of literature [3,6,31] regarding the impact of a single-risk exposure on bank systemic risk; the research into bank systemic risk is mostly focused on a particular risk exposure, and there is a lack of research on the integration of multiple-risk exposures. At the same time, most of the existing literature concerns static banking network systems, which cannot fully and accurately assess the dynamic cumulative process of risk diffusion in the banking system. In addition, most of the existing studies regarding banking systemic risk lack the support of actual data [37]. Consequently, our article discusses the impact of the integrated risk of multiple exposures of interbank lending risk, bank–entity industry credit risk, and market risk on the risk accumulation of the banking system for the first time, as well as combining geometric Brownian motion to construct a dynamically evolving banking system. In addition, this article adopts the ΔCoVaR approach instead of using the bank failure probability as the banking systemic risk measurement index and evaluates the systemic risk of Chinese banks from the perspective of risk contribution, which can more accurately reflect the process of systemic risk accumulation. Finally, the previous research on banking systemic risk lacks the support of actual data. This article collects a large amount of actual data, including the stock data of 2915 listed companies in the entity industry to estimate the dynamic credit risk of the entity industry, the yield data of five risk factors from 2007 to 2017 to calculate the market risk faced by banks, and the asset–liability data of 205 banks to estimate the risk of the interbank lending market. This amount of data is rarely studied when evaluating the banking systemic risk in China. Therefore, this study ensures a greater probability that the results will be closer to the real banking system, thus providing a good basis for subsequent modeling and a more practically meaningful output of the results.

## 3. Models and Methods

**Figure 1:**
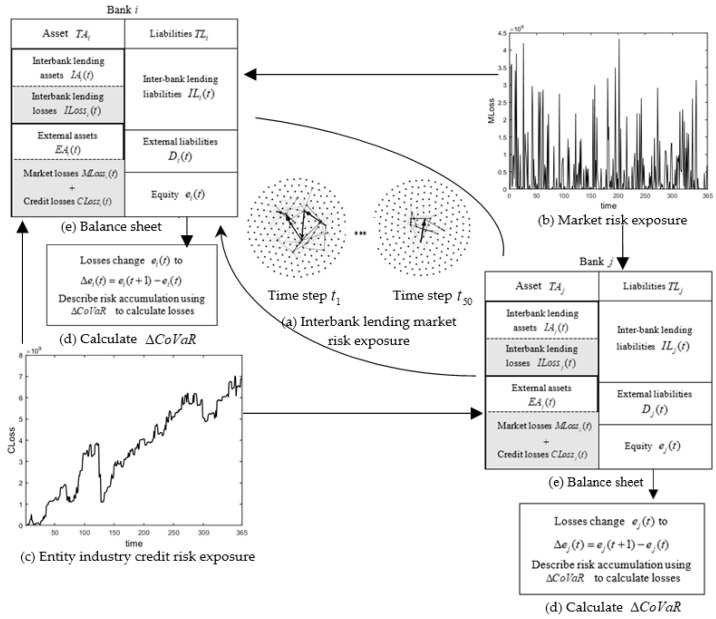
Dynamic banking systemic risk model framework under multiple exposures.

In this section, we first constructed a framework for modeling the dynamic evolution of systemic risk in banks with multiple-risk exposures, as is shown in Figure 1. Assuming that there are N banks in the banking system that all have similar balance sheet structures (as is shown in Figure 1e), the banks’ assets and liabilities evolve dynamically over time, and the risk contribution to the banking system from losses arising from different exposures also evolves dynamically. Interbank lending exists between banks, forming interbank lending market exposures, as is shown in Figure 1a. Market risk factors such as exchange rates and equities of banks are closely related to financial markets, forming market risk exposures, as is shown in Figure 1b. The credit risk exposure to the entity industry is formed by the close linkage between banks and the entity industry due to credit lending, as is shown in Figure 1c. Therefore, banks’ multiple exposures include interbank lending market exposures, entity industry credit exposures, and market exposures. In addition, the risk accumulation is described using ΔCoVaR calculated losses, as is shown in Figure 1d. Section 3.1 of this paper constructs the interbank lending market exposures corresponding to Figure 1a, Section 3.2 constructs the market exposures corresponding to Figure 1b, Section 3.3 constructs the entity industry credit exposures corresponding to Figure 1c, Section 3.4 provides an estimation of the dynamic evolution of assets and liabilities, Section 3.5 proposes the dynamic banking systemic risk evolution process with multiple exposures, and Section 3.6 discusses the systemic risk contribution ΔCoVaR model corresponding to Figure 1d.

The losses on the balance sheet in the chart are mainly from interbank lending market losses, entity industry credit losses, and market losses.

### 3.1. Interbank Lending Market Risk Exposure

**Figure 2:**
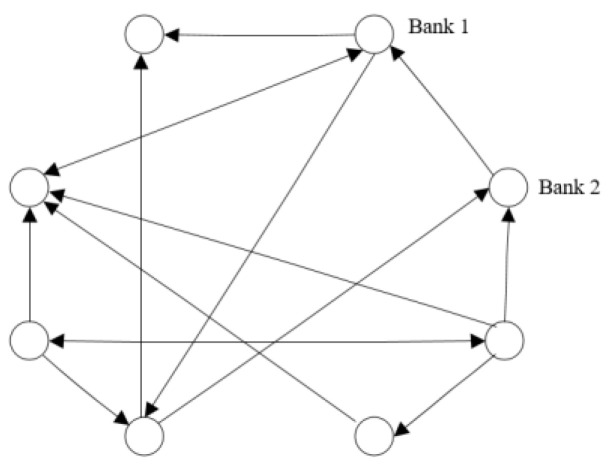
Bank network topology structure.

Banks establish a directed interbank lending network due to liquidity shortages and they form a lending relationship; the network topology is shown in Figure 2. Nodes in the network represent banks, and the directed edges between nodes represent interbank lending relationships. There are three types of lending relationships between any two bank nodes: (1) no connection means that there is no debt relationship between the two banks; (2) there is a connection and only a one-way arrow, that is, the link between the banks is from the creditor bank to the debtor bank; and (3) there is a connection and a two-way arrow, that is, the two banks are each other’s creditor bank and debtor bank.

The banking network contains a total of N banks, and X is used to describe the lending relationships between different banks in the interbank lending market, which is expressed as Equation (1). The $x_{ij}$ in Equation (1) is the flow of funds from bank i to bank j, which is an asset for bank i and liability for bank j. Since there is no self-lending between banks, $x_{ij}=0$ when i=j. The rows of the matrix are summed to obtain the total borrowing assets of bank i, denoted as $IA_{i}=\sum_{j=1}^{N}x_{ij}$, and the columns of the matrix are summed to obtain the total borrowing assets of bank j, denoted as $IL_{j}=\sum_{i=1}^{N}x_{ij}$. In addition, because the total borrowed funds in the interbank lending market are certain, it is necessary to ensure that the total borrowed assets of the banking system are equal to the total borrowed liabilities, i.e., $\sum_{i=1}^{N}IA_{i}=\sum_{j=1}^{N}IL_{j}$.

(1) $$X=\left[\begin{array}{ccccc} x_{11} & \cdots & x_{1j} & \cdots & x_{1N}\\ \vdots & \ddots & \vdots & ⋰ & \vdots \\ x_{i1} & \cdots & x_{ij} & \cdots & x_{iN}\\ \vdots & \ddots & \vdots & ⋰ & \vdots \\ x_{N1} & \cdots & x_{Nj} & \cdots & x_{NN} \end{array}\right].$$

Due to the private nature of bank transactions, specific lending data relationships between banks are difficult to obtain in practice. Therefore, there are two methods to determine the interbank lending matrix. One is to use a simulation method to simulate the interbank lending data. The other is to estimate the interbank lending matrix based on the real data of interbank assets and interbank liabilities using the maximum entropy method [38] or the minimum density method [39]. However, interbank lending behavior is inherently uncertain, and most banks are unlikely to have lending relationships with all banks in the banking system at the same time [40]. This fully connected network of banks assumed by the maximum entropy approach does not correspond to the actual banking network structure. Therefore, this paper selected the minimum density method [39] considering the matching of anisotropic and sparse connections in the banking network to estimate the interbank lending market relationship more realistically (X in Equation (1)).

The loss in the interbank lending market exposure is the loss caused by banks’ default and contagion in the banking system. According to Equation (1), when bank i is in default at time step t, bank i can only pay the repayment ratio $\chi_{i}(t)$ of the part of its debt to its creditor bank, which is the same as the Eisenberg default mechanism algorithm [41] (in Appendix A for the calculation procedure), and the interbank lending market loss $ILoss_{i}(t)=IL_{i}(t)\cdot (1-\chi_{i}(t))$ can be obtained by the repayment ratio.

### 3.2. Market Risk Exposure

Market risk for banks has also been the focus of research for a long time. The Basel Committee defines market risk as “the risk of loss of on-balance sheet and off-balance sheet positions due to market price fluctuations”, including interest rate, stock, foreign exchange, and commodity price fluctuations [42]. Banks measure their market risk by measuring the VaR value over the holding period and assuming that the market has sufficient liquidity. This paper uses the historical simulation method that does not need to assume the income distribution to measure the market risk of the bank and predicts the future income through the historical income of the market risk factors. Therefore, only the past income of market risk factors needs to be collected to analyze a bank’s potential market risk at the future time point, based on historical data.

We used $TA_{i}$ to denote the total assets of bank i, which includes interbank lending assets $IA_{i}$ and external assets $EA_{i}$. In addition, $c_{i}$ denotes the ratio of market risk factors to bank’s external assets $EA_{i}$, and $c_{iu}$ denotes the ratio of the bank’s uth market risk factor to the bank’s external assets $EA_{i}$. Thus, the market risk exposure in the banking system can be represented by matrix MRE(t):

(2) $$MRE(t)=\left(\begin{array}{ccccc} c_{11}EA_{1}(t) & \cdots & c_{1k}EA_{1}(t) & \cdots & c_{1u}EA_{1}(t)\\ \vdots & \ddots & \vdots & ⋰ & \vdots \\ c_{i1}EA_{i}(t) & \cdots & c_{ik}EA_{i}(t) & \cdots & c_{iu}EA_{i}(t)\\ \vdots & ⋰ & \vdots & \ddots & \vdots \\ c_{n1}EA_{n}(t) & \cdots & c_{nk}EA_{n}(t) & \cdots & c_{nu}EA_{n}(t) \end{array}\right).$$

A randomly selected column of income data from the history data is represented by a matrix $Y(t)=\left(y_{1}(t),y_{2}(t),\cdots y_{u}(t)\right)$, which denotes the return of the uth market risk factor at time t. Then, we multiplied the return matrix with the market risk exposure to obtain the market risk value MLoss(t), expressed in Equation (3):

(3) $$ML\mathrm{oss}(t)=\left(\begin{array}{ccccc} c_{11}EA_{1}(t)y_{1}(t) & \cdots & c_{1k}EA_{1}(t)y_{i}(t) & \cdots & c_{1u}EA_{1}(t)y_{u}(t)\\ \vdots & \ddots & \vdots & ⋰ & \vdots \\ c_{i1}EA_{i}(t)y_{1}(t) & \cdots & c_{ik}EA_{i}(t)y_{i}(t) & \cdots & c_{iu}EA_{i}(t)y_{u}(t)\\ \vdots & ⋰ & \vdots & \ddots & \vdots \\ c_{n1}EA_{n}(t)y_{1}(t) & \cdots & c_{nk}EA_{n}(t)y_{i}(t) & \cdots & c_{nu}EA_{n}(t)y_{u}(t) \end{array}\right).$$

Thus, the bank’s market loss can be expressed as ${\mathrm{MLoss}}_{i}(t)=\sum_{k=1}^{u}c_{\mathrm{ik}}{\mathrm{EA}}_{i}(t)y_{k}(t)$, when the market risk value of bank i$MLoss_{i}(t)<0$ indicates that bank i has market losses.

### 3.3. Credit Risk Exposure

The entity industry and banks are interconnected by lending relationships, forming a bank–entity industry credit network. When credit risk occurs in the entity industry due to excessive debt stress, the credit risk is transmitted through the industry–bank credit network, leading to the loss of the external assets of banks. As a result, the banking system is exposed to credit risk exposures of the entity industry. To construct a realistic bank–industry credit network, the ratio of loans to all banks in the bank–industry credit network needs to be determined. Thus, suppose there are N banks and g entity industries in the system, and the banks in the system can be classified into q types, with k banks of each type, each lending to entity industries, and banks of the same type lend the same proportion to the same type of entity industries. The average percentage of loans given by bank i to the entity industry h, under type a is denoted by $LR_{aih}=\sum_{i=1}^{k_{a}}LR_{ih}/k_{a}(a=1,2,\cdots ,q)$, where $k_{a}$ is the total number of banks under type a; $LR_{ih}=l_{ih}/EA_{i}(i=1,2,\ldots ,n;h=1,2,\ldots ,g)$ is the proportion of credit loans provided by bank i to entity industry h, where $EA_{i}$ denotes the external assets of bank i, and $l_{ih}$ denotes the loans provided by bank i to entity industry h.

The data on bank loans to the entity industry can be collected from the annual reports of banks, but it is impossible to obtain the exact percentage of loans to the entity industry from all banks. Therefore, $LR_{ih}$ is the proportion of bank loans to the entity industry that can be obtained in practice; the proportion of bank loans to the entity industry that cannot be obtained was set to the average loan proportion $LR_{qih}$ of the corresponding types of banks to the entity industry, by which the proportion matrix of all types of bank loans to the entity industry can be obtained, expressed by Equation (4):

(4) $$LR=\left(\begin{array}{ccccc} LR_{111} & \cdots & LR_{11h} & \cdots & LR_{11g}\\ \vdots & \ddots & \vdots & ⋰ & \vdots \\ LR_{qi1} & \cdots & LR_{qih} & \cdots & LR_{qig}\\ \vdots & ⋰ & \vdots & \ddots & \vdots \\ LR_{fN1} & \cdots & LR_{fNh} & \cdots & LR_{fNg} \end{array}\right).$$

When the entity industry is adversely affected and cannot fully repay its creditor banks, banks lose their external assets due to the shock of credit exposure to the entity industry. Thus, the credit exposure CRE(t) of the entity industry is the product of a bank’s external assets and credit ratio, expressed in Equation (5):

(5) $$CRE(t)=\left(\begin{array}{ccccc} EA_{1}(t)LR_{111} & \cdots & EA_{1}(t)LR_{11h} & \cdots & EA_{1}(t)LR_{11g}\\ \vdots & \ddots & \vdots & ⋰ & \vdots \\ EA_{i}(t)LR_{qi1} & \cdots & EA_{i}(t)LR_{qih} & \cdots & EA_{i}(t)LR_{qig}\\ \vdots & ⋰ & \vdots & \ddots & \vdots \\ EA_{N}(t)LR_{fN1} & \cdots & EA_{N}(t)LR_{fNh} & \cdots & EA_{N}(t)LR_{fNg} \end{array}\right).$$

We can multiply the entity industry credit exposure matrix $CRE_{}(t)$ with the entity industry default probability $p_{g}(t)$ to obtain the credit loss $CLoss_{i}(t)$ suffered by bank i due to the entity industry.

#### Calculation of Default Probability of Listed Companies in the Entity Industry

When listed companies in the entity industry are in distress, there may be a situation where the assets are less than the liabilities, and the default occurs when the listed companies in the entity industry are insolvent, which is expressed by Equation (6):

(6) $$A_{g}(t)-L_{g}(t)<0.$$

where the asset $A_{g}(t)$, liability $L_{g}(t)$ of the listed company is similarly calculated using the geometric Brownian motion and BSM option pricing model, as shown in Section 3.4.

In addition, the entity industry default status variable $SV_{g}(t)$ takes the value of 0 or 1 as an integer. If we use the default status variable $SV_{g}(t)=1$ to indicate that the entity industry listed company has defaulted, then $SV_{g}(t)$ can be expressed by Equation (7):

(7) $$SV_{g}(t)=\{\begin{array}{c} 1\\ 0 \end{array}\begin{array}{c} ifA_{g}(t)-L_{g}(t)<0\\ otherwise \end{array}.$$

Based on the calculated default state variable $SV_{g}(t)$ for the listed entity industry, for the credit default probability of the entity industry g at time step t, $p_{g}(t)$ is calculated using the Monte Carlo simulation method and expressed in Equation (8):

(8) $$p_{g}(t)=\sum SV_{g}(t)/n_{g}.$$

where $n_{g}$ is the total number of listed companies in the entity industry and $\sum SV_{g}(t)$ is the total number of all listed companies in the entity industry g that have defaulted at time step t. Furthermore, $p_{g}(t)$ is a credit default probability curve for the entity industry that varies with time step t. The larger the $p_{g}(t)$ the greater the credit default probability for the entity industry g. Conversely, the probability of credit default is smaller.

### 3.4. Estimation of the Dynamic Evolution of Assets and Liabilities

Asset values, asset value volatility, and drift rates for banks or listed companies within the entities’ industry cannot be collected directly from bank-disclosed financial statements. However, we can use the geometric Brownian motion [43] to characterize asset value fluctuations by using bank asset and liability data at the end of each year and then use equity value in the stock market and risk-free interest rates in the current market environment to derive the dynamic evolution series of liabilities, and finally estimate the time evolution series of assets. Here, the construction process of the dynamic evolution series of bank assets and liabilities is selected for specific elaboration.

To predict the external assets $EA_{i}(t)$ and external liabilities $EL_{i}(t)$ of bank i at any point in the future, it is assumed that each bank is an investor with risk-neutral characteristics and there is no risk-free arbitrage. Assuming that random changes in bank assets obey geometric Brownian motion and that each bank’s asset fluctuations are independent of each other, and allowing t to represent the asset evolution time (T is the total evolution time), we obtain Equation (9).

(9) $$dEA_{i}(t)=\mu_{i}EA_{i}dt+\sigma_{i}EA_{i}(t)dW(t),\forall i\in N,\forall t\in [0,T].$$

To obtain an accurate dynamic evolution equation for bank assets, it is necessary to estimate the drift rate and volatility parameters in the stochastic differential equation. In this paper, we referred to Equation (10) of the BSM [44] model for bank equity $e_{i}(t)$, where Θ(⋅) is the standard normal distribution and r is the risk-free rate.

(10) $$e_{i}(t)=EA_{i}(t)\Theta (dt)-EL_{i}(t)\Theta (dt-\sigma_{i}\sqrt{t}).$$

(11) $$dt=\frac{\ln(EA_{i}(t)/EL_{i}(t))+\sigma_{i}^{2}t/2}{\sigma_{i}\sqrt{t}}.$$

If all bank liabilities grow at the risk-free rate r, the dynamic evolution of bank liabilities is given by Equation (12). The unknown parameters $\mu_{i}$ and $\sigma_{i}$ in the model were estimated using the maximum likelihood estimate [45]. Bringing the bank asset drift rate $\mu_{i}$ and volatility $\sigma_{i}$ into the asset evolution equation yields Equation (13), which is the time-evolution sequence of bank assets.

(12) $$EL_{i}(t)=EL_{i}(0)e^{rt}.$$

(13) $$EA_{i}(t)=EA_{i}(0)e^{\left(\mu_{i}-(\sigma_{i}^{2}/2))t+\sigma_{i}w(t\right)}.$$

### 3.5. Dynamic Banking Systemic Risk Evolution Process under Multiple-Risk Exposures

Step 1: Estimate the evolutionary dynamics of bank assets and liabilities [21]. Based on the dynamic banking systemic risk model framework in Figure 1, we combined the geometric Brownian motion and BSM option pricing model [43,44,45] to evolve external assets $EA_{i}(t)$ and external liabilities $EL_{i}(t)$ growing at the risk-free rate for all banks at time step t. Then, we applied the minimum density method [39] to calculate the interbank lending assets $IA_{i}(t)$ and lending liabilities $IL_{i}(t)$ at time step t.

Step 2: Calculate the external asset loss $EAL{\mathrm{oss}}_{i}(t)$ at time t for bank i with multiple exposures, where the external asset loss included the market loss $MLoss_{i}(t)$ and the entity industry credit loss $CLoss_{i}(t)$. (1) Calculation of the market loss: we constructed the market exposure matrix MRE(t) in the banking system using risk factors and multiplying it by the return Y(t) of the market risk factor to obtain the market risk loss $MLoss_{i}(t)$ faced by bank i in the banking system (in Section 3.2). (2) Calculation of the credit loss of the entity industry: we constructed a matrix LR of the lending ratio between the banks and the entity industry according to the lending correlation between the entity industry and banks. When the entity industry is adversely affected, banks are hit by the credit risk exposure of the entity industry, leading to the loss of their external assets. Then, we obtained the entity industry credit risk exposure matrix CRE(t), which was multiplied by the default probability of the entity industry to obtain the credit loss $CLoss_{i}(t)$ of bank i due to the entity industry (in Section 3.3).

Step 3: Calculate the bank repayment ratio [41]. Based on the accounting constants, we calculated the equity of bank i at time step t as

(14) $$e_{i}^{}(t)=(EA_{i}(t)-MLoss_{i}(t)-CLoss_{i}(t))+(IA_{i}(t)-ILoss_{i}(t))-EL_{i}(t)-IL_{i}(t).$$

where the interbank lending asset loss $ILoss_{i}(t)$ (in Section 3.1) occurs after contagion, so the initial $ILoss_{i}(t)$ is zero. When $e_{i}^{}(t)>0$, this means that bank i can repay all the lending liabilities of its creditor banks, i.e., the repayment ratio $\chi_{i}$ is 1. When $e_{i}^{}(t)<0$, this means that bank i cannot repay all the lending liabilities of its creditor banks and the repayment ratio [41] $\chi_{i}$ of bank i is calculated (in Appendix A).

Step 4: Update the split matrix [39] based on the repayment ratio. When a bank’s repayment ratio $\chi_{i}<1$, the lending assets of the banks with which bank i has a lending relationship are updated in the lending matrix. When there is a change in the lending assets, the loss on lending between banks $ILoss_{i}(t)$ can be calculated. Since the contagion relationship does not necessarily occur sequentially, the equity of banks with lending relationships needs to be recalculated when $ILoss_{i}(t)$ changes.

Step 5: Repeat steps 1–4 to obtain the equity of bank i at time t. The formula is

(15) $$e_{i}^{}(t+1)=(EA_{i}(t+1)-MLoss_{i}(t+1)-CLoss_{i}(t+1))+(IA_{i}(t+1)-ILoss_{i}(t+1))-EL_{i}(t+1)-IL_{i}(t+1).$$

Step 6: Using Equations (9) and (10). calculate the value of loss for bank i.

(16) $$\Delta e_{i}(t)=e_{i}(t+1)-e_{i}(t).$$

when $\Delta e_{i}(t)<0$, there is loss.

### 3.6. Value-at-Risk Model

The VaR model (value at risk) is a comprehensive risk measure based on the integration of statistical and financial knowledge first proposed by Jorion [46]. The comprehensiveness of the risk measure mainly lies in the fact that the risk of financial assets can be expressed visually. As a risk measurement technique, the VaR method has now become the mainstream method for measuring market risk in the financial world, and VaR is defined specifically as the maximum loss that the value of the assets of the banking system may suffer in a certain period in the future under normal market fluctuations and a given 1−α confidence level. Its mathematical formula is expressed as follows:

(17) $${\mathrm{Pr}}_{t}(TLoss_{S}^{}(t)\leq VaR_{S}^{\alpha }(t))=\alpha .$$

where $TLoss_{S}^{}(t)=\sum_{i=1}^{n}\Delta e_{i}(t)$ is the total loss of the banking system at time step t, and $\sum_{i=1}^{n}\Delta e_{i}(t)$ is the sum of the losses of the banking system. The definition of VaR describes the quantile under the probability distribution of returns and losses within a certain period. The VaR model can only assess the value-at-risk of a single bank in an extreme market environment and does not adequately reflect the risk spillover of each bank in the banking system. Therefore, Adrian et al. [12] proposed a conditional CoVaR model based on the VaR model to solve the above problem. It is defined by the a-quantile of the conditional probability distribution, that is, $CoVaR_{s|i}^{\alpha }(t)$ represents the VaR of the banking system conditional on losses of bank i at time step t.

(18) $${\mathrm{Pr}}_{t}(TLoss_{S}^{}(t)\leq CoVaR_{s|i}^{\alpha }(t)|\Delta e_{i}(t)=VaR_{i}^{\alpha }(t))=\alpha .$$

The above equation $CoVaR_{s|i}^{\alpha }(t)$ represents the spillover risk of bank i to the banking system, and CoVaR can also be considered as special VaR. Therefore, the risk contribution of a single bank to the banking system can be expressed as the difference between the risk of the system $CoVaR_{s|i}^{\alpha }(t)$ and the risk of the banking system in normal times $CoVaR_{s|i}^{median}(t)$, i.e., the spillover value-at-risk $\Delta CoVaR_{s|i}^{\alpha }(t)$:

(19) $$\Delta CoVaR_{s|i}^{\alpha }(t)=CoVaR_{s|i}^{\alpha }(t)-CoVaR_{s|i}^{median}(t)=\gamma (t)\cdot (VaR_{i}^{\alpha }(t)-VaR_{i}^{median}(t)).$$

where γ(t) is the correlation coefficient between bank i and the banking system at time step t. The quantile regression derivation yields that the correlation coefficient γ(t) of bank i with the banking system multiplied by the difference between bank i in a crisis period and a normal state is the risk spillover value of bank i to the banking system, also known as the risk contribution of bank i to the banking system. In this way, the risk contribution of the entity industry to the system and the risk contribution of the market risk factor to the banking system can also be calculated.

## 4. Results

### 4.1. Data

This study uses real data from Chinese banks, information on listed companies, and market risk factors. There are three parts to the real data obtained: the first part is the data related to the public disclosure of major banks in China at the end of 2016, obtained from the CSMAR economic and financial database, and the asset and liability and lending asset and liability data of 205 banks were selected. The descriptive statistics table of these banks’ assets, liabilities, lending assets, and lending liabilities data are shown in Table 1. As is shown in Table 1, the standard deviation is higher than the mean, indicating that there was a large divergence in bank asset–liability data. In addition, the mean value of interbank lending was much higher than the median, which means that data with larger lending amounts were skewed.

The second part is obtained from the CSMAR economic and financial database of the 2012 revised industry classification of listed companies by the China Securities Regulatory Commission, and the data of assets and liabilities as well as 244 days of individual stock trading amounts for a total of 2915 listed companies in 18 industries that were selected. The statistical table of the data from these listed companies’ is shown in Table 2. Among them, the manufacturing industry had the largest number of listed companies, accounting for 62.9% of the overall sample number, and the residential service industry and education industry had the least amount, accounting for 0.1% of the overall sample number.

In the table, IB indicates the entity industry name abbreviations, the table in Appendix C has entity industry name abbreviations corresponding to the table, Qty indicates the number of entity industries, and % indicates the proportion of an entity industry to all industries.

**Figure 3:**
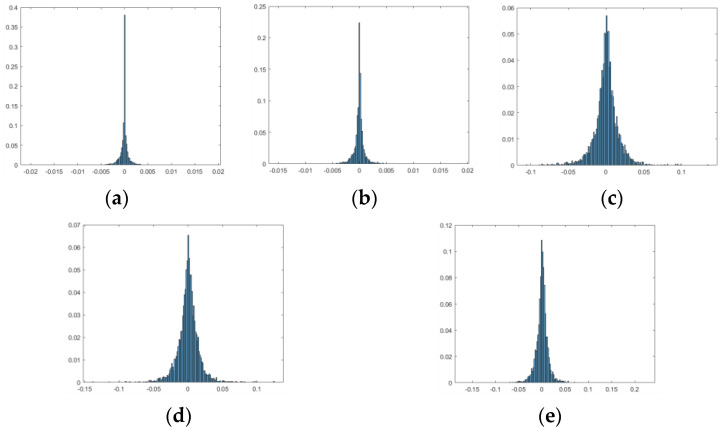
Market risk factor return distribution chart. (The horizontal coordinate represents the rate of return, and the vertical coordinate represents the probability; (**a**) represents the return of USD/CNH; (**b**) represents the return of HKD/CNH; (**c**) represents the return of SSE Composite Index; (**d**) represents the return of Hang Seng Composite Index; and (**e**) represents the return of Dow Jones Index.).

The third part consists of the market risk factor data obtained from the wind economic database. The trading day data from 2007–2017 for five market risk factors, namely the USD/CNH exchange rate, the HKD/CNH exchange rate, the SSE Composite Index, the Hang Seng Composite Index, and the Dow Jones Index, were selected. The probability distribution functions of returns for these five market risk factors are shown in Figure 3. According to the distribution chart, it can be seen that the returns of the two foreign currencies of USD/CNH and HKD/CNH among the five market risk factors are approximately −0.005 to 0.005, which means that they were less volatile. The returns of three stocks, the SSE Composite Index, Hang Seng Composite Index, and Dow Jones Index, are more similar, between −0.1 and 0.1, which means that they were more volatile.

Before using the collected data, some processing was also required. First, in the estimation of interbank lending market risk, the total lending assets and liabilities of the banking system must be equal. To satisfy this feature, we used a dummy bank to absorb the excess lending assets and liabilities and maintain the lending balance of the banking system. Calculation of the dynamic bank asset-liability series $EA_{i}(t)$$EL_{i}(t)$ using geometric Brownian motion, the BSM model, and the minimum density method estimated the interbank lending matrix X to obtain interbank lending assets $IA_{i}(t)$ and lending liabilities $IL_{i}(t)$. Then, based on the repayment ratio $\chi_{i}(t)$, the interbank lending loss $ILoss_{i}(t)$ was calculated. Second, the collected industry stock data had a small number of industries with missing individual stock trading amounts for listed companies on a certain day or days, which were uniformly filled with the mean value. In addition, stress tests were conducted on the listed companies to make them default on credit with a 30% asset loss shock. Next, the credit risk loss $CLoss_{i}(t)$ was calculated based on the probability of default $p_{g}(t)$ and the exposure matrix CRE(t). Finally, some of the transaction data of market risk factors were daily data and some were weekday data; we unified them as weekday data and deleted the redundant data. Since it is difficult to obtain how many assets of banks are exposed to market risk, this paper set 5% of assets in the banking system with market risk. Then, the market risk loss $MLoss_{i}(t)$ was calculated based on the market risk exposure matrix MRE(t) and the market risk factor return Y(t). For the convenience of representation, the 205 banks in this paper were denoted by the numbers 1–205, and the 18 industries were denoted by the numbers 1–18 (the names of banks and entity industries are listed in Appendix B and Appendix C).

### 4.2. Banking Systemic Risk Analysis

**Figure 4:**
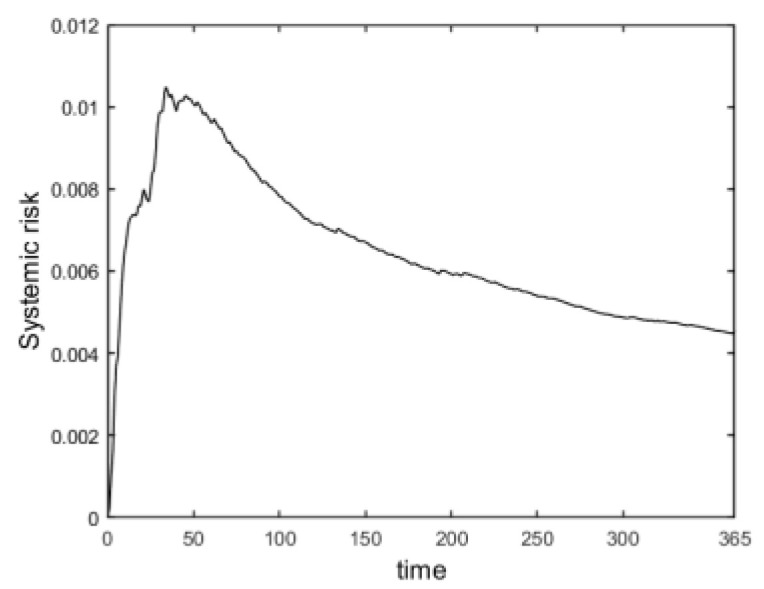
Dynamically evolving cumulative banking systemic risk.

**Figure 5:**
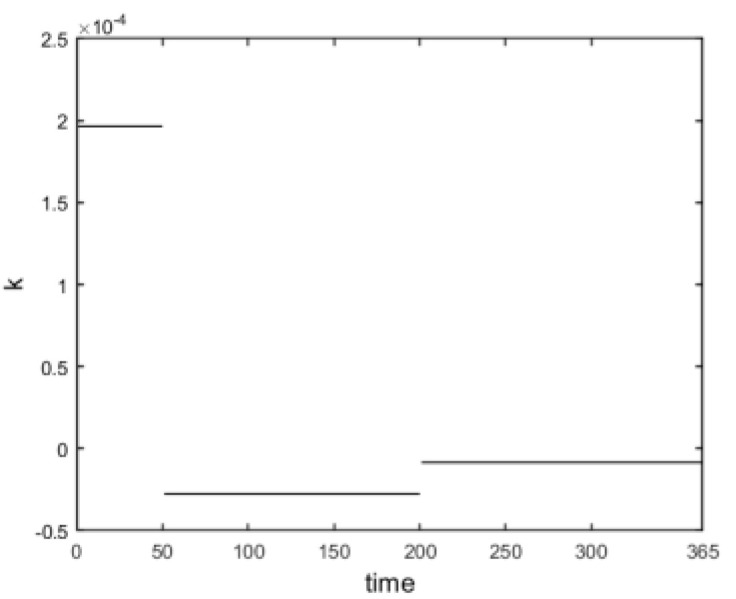
Rate of change in banking systemic risk.

In this paper, Monte Carlo simulation was performed 1000 times in calculating systemic losses, and the sum of all bank losses of the system at the smallest 5% of each time step was selected by the standard historical simulation method at a 95% confidence level, with an expected return value of 0 according to Equation (17). The ratio of total losses to the net assets of the banking system was used to measure the systemic risk and the cumulative systemic risk of the banking system over time, as is shown in Figure 4. The number of defaulting banks at each time step was recorded while calculating bank losses; at the same time, the derivative of the calculated dynamic systemic risk value was obtained to observe the rate of change in banking systemic risk, as is shown in Figure 5.

From the overall view of the curve in Figure 4, the systemic risk of banks was accumulating as the time step increased; then, the systemic risk gradually decreased. Combined with the rate of change in banking systemic risk in Figure 5, specifically, in the first 50 time steps (early period), 80 banks defaulted. The banking system underwent large shocks and kept generating losses, and the systemic risk accumulated sharply and rose, reaching the maximum at the time when the rate of change in bank systemic risk was the largest (the first 50 time steps, as is shown in Figure 4 and Figure 5). As the time step progressed from the 50th to the 200th time step (mid-term), the number of banks defaulting in the system decreased to 24, and many banks defaulting in the earlier period caused the systemic risk to be released. In addition, the systemic risk gradually decreased and the rate of accumulation of banking systemic risk slowed down (50–200 time steps, as is shown in Figure 4 and Figure 5). Finally, in the 200th to 365th time step (late), only 10 banks defaulted in the banking system, and the rate of change in the banking systemic risk approached 0, which was slower compared to the previous period, as the system gradually stopped having bank defaults and plateaus (200–365 time steps, as is shown in Figure 4 and Figure 5).

### 4.3. Cumulative Analysis of Different Risk Exposures to the Banking Systemic Risk

**Figure 6:**
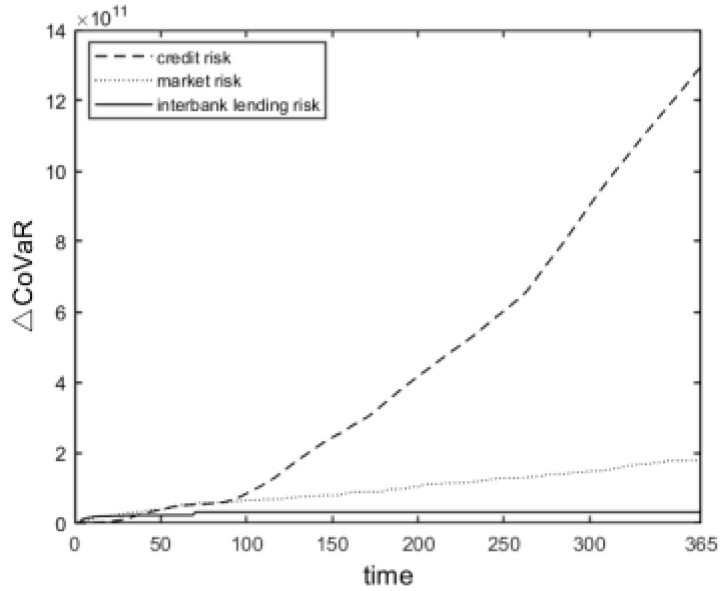
Contribution of different risk exposures to the accumulation of banking systemic risk.

As mentioned earlier, systemic risk accumulation consists of the accumulation of interbank lending market exposures, market exposures, and credit exposures. As time advances, the contribution of each exposure in the banking system to the accumulation of systemic risk in banks changes, as is shown in Figure 6 (the solid line indicates the contribution of interbank lending market exposures to the accumulation of systemic risk; the dashed line indicates the contribution of market exposures to the accumulation of systemic risk; and the double-dashed line indicates the contribution of credit exposures to the accumulation of systemic risk). All exposures are dynamically changing in their contribution to systemic risk, and each step of change represents a day with differences in the degree of change in the cumulative contribution to systemic risk of banks for different exposures.

All three exposure types showed an increasing trend in their cumulative contribution to systemic risk, but the increasing trend differed in different periods. Comparing the three curves in Figure 6, we can see that the cumulative contribution of all three exposures to systemic risk increased in the early period, and market exposures and entity industry credit exposures contributed more to the cumulative systemic risk. The cumulative contribution of interbank lending market exposures to systemic risk tended to stabilize in the medium term, and the cumulative contribution of market exposures to systemic risk increased to a lesser extent, while the cumulative contribution of credit exposures to systemic risk tended to increase significantly and contributed the most. In the later period, similar to the medium-term period, the contribution of market exposures to the accumulation of systemic risk increased to a lesser extent, and the accumulation of systemic risk mainly stemmed from the accumulation of credit exposures to systemic risk. Overall, the contribution of the three exposures to the accumulation of systemic risk was as follows: entity industry credit exposures > market exposures > interbank lending market exposures. When regulators supervise banks’ systemic risk, they should keep abreast of the changes in systemic risk due to different exposures, regularly observe the accumulation of risk to the system from different exposures, and reasonably formulate and adjust macroprudential supervision policies for systemic risk from different exposures in a timely manner.

### 4.4. Analysis of the Cumulative Contribution of Interbank Lending Market Risk to Systemic Risk

**Figure 7:**
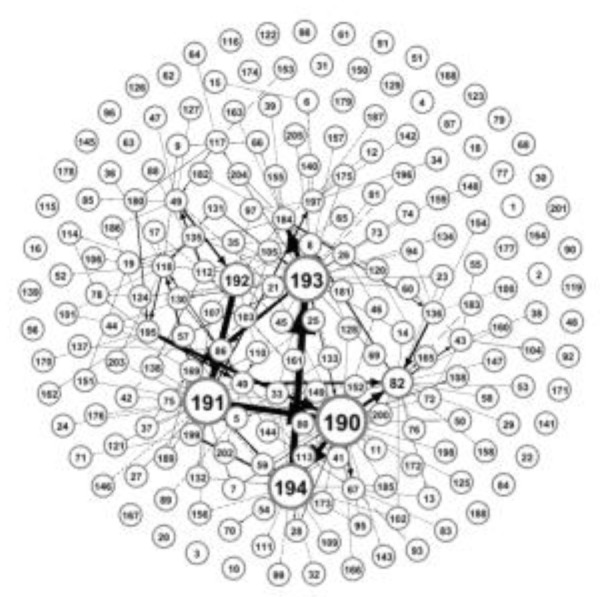
Interbank network structure.

The close ties between banks due to lending relationships also provide a channel for risk contagion. Some banks in the banking system experience financial distress due to their operating conditions, meaning that their debts will not be fully repaid to their creditor banks, thus adversely affecting them through interbank lending market exposures, and leading to losses in creditor banks’ assets. We estimated the interbank lending matrix to draw a directed graph of the interbank market network structure, as shown in Figure 7. The serial number in the nodes is the serial number of the bank (the bank name corresponding to the serial number is in Appendix B); the size of the nodes indicates the degree of connectivity, and the larger the nodes, the greater the connectivity. The directed connections between the nodes indicate the interbank lending relationship, and the thickness of the connections indicates the size of the lending amount; the thicker the connections, the more the lending amount, the size, and the number of nodes, and the thickness of the connections in the figure will change over time. The top four banks in terms of connectivity are the Industrial and Commercial Bank of China (190), the China Construction Bank (191), and the Agricultural Bank of China (193), and the thickest line between these banks is the line from 194 to 193, indicating that the Bank of China (194) lends the most money to the Agricultural Bank of China (193).

**Figure 8:**
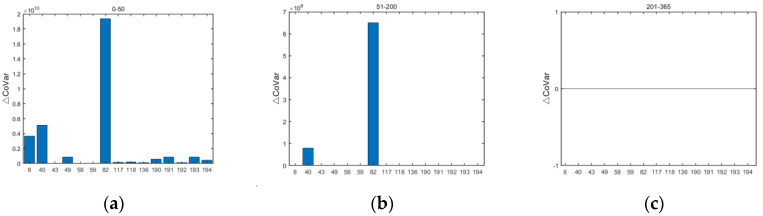
Contribution of interbank lending market risk to systemic risk accumulation in different periods. (The horizontal coordinate represents the bank serial number, the vertical coordinate represents the contribution to systemic risk accumulation, and the three panels represent the sum of the contribution to risk accumulation in the three time spans of (**a**) early (0–50 time steps), (**b**) middle (51–200 time steps), and (**c**) late (201–365 time steps)).

While the lending relationship gives an indication of the size of interbank lending, it does not discern the extent to which banks contribute to systemic risk. Hence, identifying which banks in the system contribute significantly to the accumulation of systemic risk due to interbank lending market risk in different periods helps regulators monitor risk contagion among banks. The contribution of interbank lending market risk to systemic risk accumulation in different periods is shown in Figure 8.

Interbank lending market risk contributes to systemic risk accumulation due to risk contagion from bank defaults in the banking system. The contribution of interbank lending market risk to systemic risk accumulation fluctuates and decreases with the increase in time step, until the later period when no banks generate interbank lending market risk due to risk contagion and the contribution to bank systemic risk accumulation stabilizes. The Bank of Communications (82) and Everbright Bank (40) have been ranked the top two in terms of the contribution of interbank lending market risk to systemic risk accumulation. The Bank of Communications is a large-scale, state-controlled commercial bank, and it is a major fund splitter in interbank lending relationships, which is more likely to generate interbank lending market risk to the system. Everbright Bank (40) has developed rapidly in recent years, actively transforming and innovating, with enhanced instability and uncertainty. The interbank lending market risk contributed by the Bank of Beijing (8) to the system in the early stage should not be ignored. The interbank lending assets of these three banks accounted for about 5% of their total assets, and the more funds they withdraw, the greater the interbank lending market risk and the greater their contribution to the accumulation of systemic risk. Regulators should focus on these three banks in their supervision of the interbank lending market.

### 4.5. Analysis of the Cumulative Contribution of Market Risk to Systemic Risk

**Figure 9:**
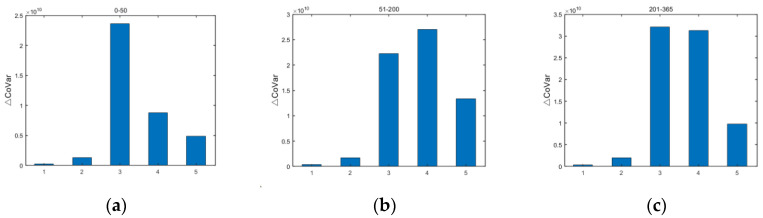
Contribution of market risk to systemic risk accumulation in different periods. (The horizontal coordinates represent the serial numbers of the five market risk factors: (1) US dollar against RMB; (2) Hong Kong dollar against RMB; (3) SSE Composite Index; (4) Hang Seng Composite Index; and (5) Dow Jones Index. The vertical coordinates represent the contribution to systemic risk accumulation, and the three plots are for the three periods of (**a**) early (0–50 time steps), (**b**) middle (51–200 time steps), and (**c**) late (201–365 time steps) and the sum of the cumulative contribution to risk over the time horizon).

A series of major risk events in the 1990s made the Basel Committee aware of the importance of market risk, and in 1996, the Basel Committee introduced amendments to the Capital Accord on market risk. Subsequently, more and more scholars have focused on the contribution of market risk to systemic risk. The direct impact of market risk on the contribution of banks to systemic risk is twofold. On the one hand, it is the volatility of market risk factor returns, and the collective returns of the five risk factors clearly show that the returns of equities are more volatile. On the other hand, it is the impact of market risk exposures, thus exploring the contribution of market risk exposures to the accumulation of systemic risk in banks. The contribution of the five market risk factors to the accumulation of systemic risk over time is shown in Figure 9.

Market risk can occur at any time and is one of the sources of risk in the banking system that cannot be underestimated. The contribution of the five risk factors to the accumulation of systemic risk varies little from period to period, but equities contribute much more to the accumulation of systemic risk than exchange rates over the same period. The greater contribution of equities to systemic risk accumulation is because equities vibrate more destructively and tend to be more susceptible to various factors such as macroeconomics, policy guidance, and market sentiment, any of which are highly uncertain; this uncertainty is the source of risk accumulation. Regulators should pay extra attention to the impact of equities on the accumulation of systemic risk when regulating market risk.

### 4.6. Analysis of the Cumulative Contribution of Entity Industry Credit Risk to Systemic Risk

**Figure 10:**
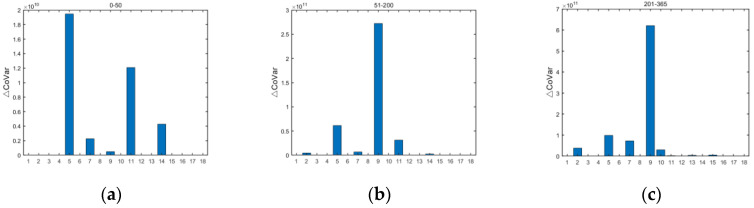
Contribution of the entity industry credit risk to systemic risk accumulation in different periods. (The horizontal coordinates represent the entity industry serial number, the vertical coordinate represents the cumulative contribution to systemic risk, and the three panels represent the sum of the cumulative contribution to risk over three time spans: (**a**) early (0–50 time steps), (**b**) mid-term (51–200 time steps), and (**c**) late (201–365 time steps)).

Different entity industries have different developments, and certain entity industries may experience drastic fluctuations due to changes in policies, natural disasters, and other factors. Once a credit default occurs in the entity industry due to external factors, it will result in the loss of the external assets of banks with which the entity industry has financial dealings, triggering systemic risk. Therefore, identifying the entity industries with high contributions to the accumulation of systemic risk in different periods helps to analyze the systemic risk of banks. The credit risk of the entity industry fluctuates continuously with the evolution of time. Firstly, we used the stock data of the entity industry to calculate the change in losses of the entity industry at different time steps, and then used ΔCoVaR to analyze the size of the contribution of the credit risk exposure of the entity industry to the accumulation of systemic risk. The contribution of the credit risk of the 18 entity industries to the accumulation of systemic risk at different periods is shown in Figure 10. 

The risk contribution of different entity industries to the system varies from period to period, and the peak risk contribution of entity industries to the system increases as the time step progresses. The entity industries with a high credit risk contribution in the early period were accommodation and catering (5), wholesale and retail trade (11), and agriculture, forestry, and fishery (14); the entity industries with a high credit risk contribution in the middle period were finance (9), accommodation and catering (5), and wholesale and retail trade (11); and the entity industries with a high credit risk contribution in the late period were finance (9), accommodation and catering (5), and manufacturing (7). The remaining entity industries contributed less to the credit risk of the system. Combining all time steps and peak changes in general, the finance, accommodation and catering, and manufacturing industries were the three entity industries that contributed most to the systemic risk.

The financial industry is inherently a high-risk industry characterized by highly indebted operations and efficiency dependence, and the accommodation and catering industry is constantly fluctuating in demand as a result of changes in how people gather and travel; therefore, these are industries with a high credit risk contribution. The manufacturing industry has an important share of the economy. When overcapacity in the manufacturing industry puts pressure on producer prices, it is difficult for enterprises to make profits and the possibility of difficulties in recovering funds increases. At the same time, the technology development and renewal of the manufacturing industry also require a large amount of capital investment, thus contributing to the risk of the system. It is suggested that regulators should focus on the entity industries with a high credit risk contribution in different periods and allocate credit resources reasonably.

## 5. Conclusions

Banks occupy a major position in the modern economic system and are an indispensable medium for its smooth functioning. Therefore, the study of bank systemic risk is of great significance. Current research regarding bank systemic risk focuses on the impact of single exposures on bank systemic risk. Research into common exposures is still lacking and does not correspond to the actual situation. Although some of the studies exploring common exposures have enriched the study of bank systemic risk, bank assets and liabilities are mostly static, and only the impact of banks’ corporate two-tier network on systemic risk has been explored, which lacks the important risk transmission channel of the market exposures faced by banks. At the same time, the lack of data also makes current research results limited and one-sided. In summary, this paper integrated interbank lending market exposures, market exposures, and entity industry credit exposures under the same framework and used actual data from China to study the accumulation of systemic risk in banks under the dynamic evolution of multiple exposures. First, through geometric Brownian motion, the BSM model evolved the dynamic assets and liabilities of banks and constructed three exposures and calculated the banks’ total loss. Then, the contributions of different exposures to banking systemic risk accumulation were analyzed in the framework of multiple-risk exposures with the historical simulation ΔCoVaR method. Finally, the dynamic evolution of Chinese banking systemic risk accumulation under multiple-risk exposures was analyzed using Chinese stock data of entity industries, bank asset and liability data, and market risk factor data. Specifically, the following conclusions were obtained.

Over time, the systemic risk of banks accumulates and then decreases, and the contribution of different exposures to the accumulation of banking systemic risk varies. In the first period, the accumulation rate of systemic risk rose sharply, and market exposures and entity industry credit exposures contributed more to the systemic risk. In the middle period, the accumulation rate of banking systemic risk declined, and the contribution of interbank lending market exposure and market exposure to systemic risk accumulation stabilized, while the contribution of entity industry credit risk exposure to systemic risk accumulation was the largest at this time. Banking systemic risk continued to decline and level off in the later period. Similar to the medium-term period, systemic risk accumulation mainly stemmed from the contribution of entity industry credit exposures to systemic risk accumulation. Overall, entity industry credit exposures are the exposures with the largest impact on systemic risk accumulation among the three exposures. Thus, regulators should focus on credit risk exposures to guard against adverse shocks to the financial system from the entity industry.

The banks with the largest contribution to systemic risk accumulation from interbank lending market exposures were the Bank of Communications, Everbright Bank, and Bank of Beijing. The entity industries with the largest contribution to systemic risk accumulation from entity industry credit exposures were finance, accommodation and catering, and manufacturing. The three stock market risk factors that contributed the most to the accumulation of systematic risk were the SSE Composite Index, the Hang Seng Index, and the Dow Jones Index. The impact of different exposures on banking systemic risk accumulation varies across time, and regulators need to pay special attention to banks, entity industries, and market risk factors with a high contribution to risk accumulation to prevent widespread risk contagion caused by small events. Based on this, this article makes the following recommendations: (1) Regulators should focus on risk prevention, strengthen supervision of joint-stock commercial banks, and urge them to improve their internal control mechanisms. (2) Risk management of enterprises with high leverage and related to national economic life should be strengthened, and credit resources allocated reasonably. (3) The identification, assessment, and control of stock market risks should be improved.

The results of this paper will enrich the research perspective of systemic risk in banks and provide a corresponding basis for banks to prevent and monitor systemic risk. However, Chinese bank risks are not limited to the three risk scenarios considered in this paper, and the cascading contagion of risks due to excessive connections with certain industries is also well worth exploring. In addition, with the application and development of information technology in the financial industry, operational risk is also one of the risks that banks cannot ignore, and the addition of operational risk will make the study of systemic risk in banks more complete. In particular, the control of the stability of the system is also a challenge worth tackling.

## Figures and Tables

**Table 1:** Descriptive Statistics of Assets, Liabilities, Lending Assets, and Lending Liabilities of the Banking System (unit: $\times 10^{10}$ yuan).

	Mean	SD	Max	Med	Min	Tol
Assets	86.18	291.19	2220.98	10.18	0.27	17,667.22
Liabilities	80.04	269.16	2040.93	9.57	0.65	16,408.82
Lending Assets	2.01	6.90	50.43	0.014	0	410.28
Lending Liabilities	1.44	5.44	47.76	0.041	0	294.57

**Table 2:** Statistics of listed companies in 18 entity industries.

IB	Qty	%	IB	Qty	%	IB	Qty	%
MFG	1834	62.9	REI	134	4.6	FIN	77	2.6
ITS	217	7.4	TSP	96	3.3	MI	75	2.6
SRT	158	5.4	CON	87	3.0	RBS	46	1.6
CSE	44	1.5	WR	32	1.1	HSW	7	0.2
AFF	37	1.3	CI	20	0.7	EDU	3	0.1
WEP	36	1.2	AC	11	0.4	RRO	3	0.1
Tol			2915			100%		

## Data Availability

All data used to support this study are in the appendix.

## Appendix A. Calculation of Repayment Ratio

Bank i may also experience a contagion default because its debtor bank is unable to repay all its interbank borrowings. Here, we normalize the interbank lending market exposures to obtain a new matrix $\pi_{ji}(t)$ as in Equation (A1):

(A1) $$\pi_{ji}(t)=\{\begin{array}{cc} \frac{x_{ji}(t)}{IL_{i}(t)} & IL_{i}(t)>0\\ 0 & otherwise \end{array}$$

where $IL_{i}(t)=\sum_{j=1}^{N}x_{ji}(t)$ is the total debit liability of bank i at time t. The clearing vector mechanism proposed by Eisenberg and Noe [41] clears each bank through the $p_{i}^{*}(t)$ clearing vector, as in Equation (A2):

(A2) $$p_{i}^{*}(t)=\{\begin{array}{cc} IL_{i}(t) & \sum_{j=1}^{N}{\prod^{\prime }}_{ij}(t)p_{j}^{*}(t)+e_{i}(t)\geq IL_{i}(t)\\ \sum_{j=1}^{N}{\prod^{\prime }}_{ij}(t)p_{j}^{*}(t)+e_{i}(t) & 0\leq \sum_{j=1}^{N}{\prod^{\prime }}_{ij}(t)p_{j}^{*}(t)+e_{i}(t)<IL_{i}(t)\\ 0 & \sum_{j=1}^{N}{\prod^{\prime }}_{ij}(t)p_{j}^{*}(t)+e_{i}(t)<0 \end{array}$$

In Equation (A2), if bank i’s interbank lending assets $\sum_{j=1}^{N}{\prod^{\prime }}_{ij}(t)p_{j}^{*}(t)$ and equity $e_{i}(t)$ are greater than its total interbank lending liabilities $IL_{i}(t)$, then the bank can repay, and the bank’s debt payment is $IL_{i}(t)$; if the sum of bank i’s interbank lending assets $\sum_{j=1}^{N}{\prod^{\prime }}_{ij}(t)p_{j}^{*}(t)$ and equity $e_{i}(t)$ is greater than 0 and less than its total interbank lending liabilities $IL_{i}(t)$, then the bank is in default and the ability to repay is $\sum_{j=1}^{N}{\prod^{\prime }}_{ij}(t)p_{j}^{*}(t)+e_{i}(t)$; if the sum of interbank lending assets and equity of bank *i* is less than 0, then the bank is also in default and is insolvent.

The Eisenberg default mechanism algorithm is used to find $p_{i}^{*}(t)$. It is worth noting that the repayment to its debtor bank in case of bank i’s default is limited, and the repayment ratio is set to $\chi_{i}(t)$, expressed by Equation (A3):

(A3) $$\chi_{i}(t)=\frac{\sum_{j=1}^{N}\pi_{ij}(t)p_{j}^{*}(t)+e_{i}(t)}{IL_{i}(t)}$$

## Appendix B. List of 205 Banks

**No.**	**Name of Bank**	**No.**	**Name of Bank**
1	Anhui Qingyang Rural Commercial Bank	104	JPMorgan Chase Bank (China)
2	Anhui Tongcheng Rural Commercial Bank	105	Nanchong City Commercial Bank
3	Anqing Rural Commercial Bank	106	Nanhai Rural Commercial Bank
4	Bank of Anshan	107	Bank of Nanjing
5	ANZ (China)	108	South Business (China)
6	Contractor Bank	109	Bank of Inner Mongolia
7	Beijing Rural Commercial Bank	110	Bank of Ningbo
8	Bank of Beijing	111	Bank of Ningxia
9	Bank of Bohai	112	Agricultural Development Bank
10	Bank of Cangzhou	113	Panzhihua Commercial Bank
11	Bank of Changshu	114	Pangu China
12	Chaoyang Bank	115	Peixian Rural Commercial Bank
13	Chengdu Rural Commercial Bank	116	Pengcheng Rural Commercial Bank
14	Bank of Chengdu	117	Ping An Bank
15	Bank of Chengde	118	Pudong Development Bank
16	Chizhou Jiuhua Rural Commercial Bank	119	Qilu Bank
17	Cixi Rural Commercial Bank	120	Qi Shang Bank
18	Bank of Dazhou	121	Corporate Banking (China)
19	Dahua Bank (China)	122	Qidong Rural Commercial Bank
20	Dalian Rural Commercial Bank	123	Qinhuangdao Bank
21	Bank of Dalian	124	Bank of Qingdao
22	Datong Bank	125	Bank of Qinghai
23	Bank of Dandong	126	Qujing Commercial Bank
24	Deqing Rural Commercial Bank	127	Bank of Quanzhou
25	Great Wall Huaxi bank	128	Rizhao Bank
26	Deutsche Bank China	129	Swiss Bank
27	Bank of Dongguan	130	Mizuho Bank
28	East Asia China	131	Sumitomo Mitsui (China)
29	Bank of Dongying	132	Xiamen International Bank
30	Ordos Rural Commercial Bank	133	Xiamen Rural Commercial Bank
31	Feixi Rural Commercial Bank	134	Xiamen Bank
32	Foshan Rural Commercial Bank	135	Shanghai Rural Commercial Bank
33	Fujian Strait Bank	136	Bank of Shanghai
34	Fujian Nan’an Rural Cooperative Bank	137	Bank of Shaoxing
35	Fujian Zhangzhou Rural Commercial Bank	138	Shengjing Bank
36	Bank of Fuxin	139	Shizuishan Bank
37	Fubon bank	140	Shucheng Rural Commercial Bank
38	Fudian Bank	141	Shuyang Rural Commercial Bank
39	Fuyang Rural Commercial Bank	142	Shunde Rural Commercial Bank
40	China Everbright Bank	143	Sichuan Tianfu Bank
41	Guangdong Huaxing Bank	144	Bank of Suzhou
42	Guangdong Nanyue Bank	145	Suining Rural Commercial Bank
43	Guangdong Development Bank	146	Bank of Suining
44	Guangxi Beibu Gulf Bank	147	Bank of Taizhou
45	Guangzhou Rural Commercial Bank	148	Taicang Rural Commercial Bank
46	Bank of Guangzhou	149	Bank of Tai’an
47	Bank of Guiyang	150	Bank of Tangshan
48	Guilin Bank	151	Tianjin Rural Commercial Bank
49	China Development Bank	152	Bank of Tianjin
50	National (China)	153	Weihai Commercial Bank
51	Harbin Bank	154	Bank of Weifang
52	Haian Rural Commercial Bank	155	Wenling Rural Commercial Bank
53	Bank of Handan	156	Bank of Wenzhou
54	Hanya Bank (China) Co., Ltd.	157	Bank of Wuhai
55	HanKou Bank	158	Bank of Urumqi
56	Hangzhou United Bank	159	Wuxi Rural Commercial Bank
57	Bank of Hangzhou	160	Wujiang Rural Commercial Bank
58	Hefei Science and Technology Rural Commercial Bank	161	Wuhan Rural Commercial Bank
59	Bank of Hebei	162	Bank of Xi’an
60	Heng Feng Bank	163	Xining Rural Commercial Bank
61	Bank of Hengshui	164	Xiaoshan Rural Commercial Bank
62	Huludao Bank	165	New Korea China
63	Bank of Hubei	166	Xinhui Rural Commercial Bank
64	Bank of Huzhou	167	Xinyi Rural Commercial Bank
65	Huarong Xiangjiang Bank	168	Bank of Xingtai
66	Bank of China	169	Industrial Bank
67	Huaxia Bank	170	Yantai Bank
68	Huanghe Rural Commercial Bank	171	Yibin Commercial Bank
69	Huishang Bank	172	Yiwu Rural Commercial Bank
70	HSBC China	173	Yinzhou Bank
71	Bank of Jilin	174	Bank of Yingkou
72	Bank of Jining	175	Youli (China)
73	Jiangdu Rural Commercial Bank	176	Yuhang Rural Commercial Bank
74	Jiangmen Ronghe Rural Commercial Bank	177	Yuhuan Rural Commercial Bank
75	Jiangnan Rural Commercial Bank	178	Yuexi Rural Commercial Bank
76	Jiangsu Jiangyin Rural Commercial Bank	179	Yunnan Hongta Bank
77	Jiangsu Suining Rural Commercial Bank	180	Standard Chartered China
78	Bank of Jiangsu	181	Zhangjia Hong Kong
79	Jiangsu Changjiang Commercial Bank	182	Bank of Changan
80	Bank of Jiangyin	183	Bank of Changsha
81	Jiangyan Rural Commercial Bank	184	China Merchants Bank
82	Bank of Communications	185	Zhejiang Chouzhou Commercial Bank
83	Jiaojiang Rural Cooperative Bank	186	Zhejiang Mintai Commercial Bank
84	Bank of Jinhua	187	Zhejiang Tailong Commercial Bank
85	Bank of Jinzhou	188	Zhejiang Merchants Bank
86	Export Import Bank	189	Bank of Zhengzhou
87	Bank of Jincheng	190	Industrial and Commercia Bank of China
88	Jinjiang Rural Commercial Bank	191	China Construction Bank
89	Shanxi Merchants Bank	192	China Minsheng Bank
90	Jingxian Rural Commercial Bank	193	The Agricultural Bank of China
91	Jingdezhen Rural Commercial Bank	194	Bank of China
92	Bank of Jiujiang	195	China Post Savings Bank
93	Bank of Kunlun	196	Zhongshan Rural Commercial Bank
94	Kunshan Rural Commercial Bank	197	CITIC Bank
95	Lai Hang Bank	198	Zhongyuan Bank
96	Bank of Lanzhou	199	Chongqing Rural Commercial Bank
97	Leshan Commercial Bank	200	Chongqing Three Gorges bank
98	Liangshan Commercial Bank	201	Bank of Chongqing
99	Pro Bank	202	Zhuhai China Resources Bank
100	Bank of Liuzhou	203	Zhuhai Rural Commercial Bank
101	Bank of Longjiang	204	Zijin Rural Commercial Bank
102	Luzhou Commercial Bank	205	Bank of Zigong
103	Bank of Luoyang		

## Appendix C. List of 18 Entity Industries

**No.**	**Name of Industry**	**Abbreviation**
1	Water, environment, and public facilities management industry	WEP
2	Mining industry	MI
3	Residential services, repairs, and other services	RRO
4	Transportation, storage, and postal industry	TSP
5	Accommodation and catering	AC
6	Scientific research technology services	SRT
7	Manufacturing	MFG
8	Construction	CON
9	Finance	FIN
11	Wholesale and retail trade	WR
12	Rental and business services	RBS
13	Information transmission, software, and information technology services	ITS
14	Agriculture, forestry, and fisheries	AFF
15	Culture, sports, and entertainment	CSE
16	Health and social work	HSW
17	Education	EDU
18	Comprehensive industry	CI
